# Supraglottic Neuroendocrine Carcinoma: A Case Report and Literature Review

**DOI:** 10.1155/2024/6844193

**Published:** 2024-10-01

**Authors:** Renda Alhabib, Chaker Zaidi, Abdulrahman Alfryyan, Hanan Albenayyan, Ibrahim Alotain

**Affiliations:** ^1^ Radiation Oncology Department of Oncology King Fahad Specialist Hospital, Dammam, Saudi Arabia; ^2^ Imam Abdulrahman Bin Faisal University, Dammam, Saudi Arabia; ^3^ General Pediatrics Department King Fahad University Hospital, Khobar, Saudi Arabia

**Keywords:** atypical carcinoid, neoplasms of the larynx, neuroendocrine carcinoma

## Abstract

Neuroendocrine neoplasms (NENs) are rare in the head and neck region, with the larynx being the most common site. To date, nearly 700 cases of laryngeal neuroendocrine carcinoma (NEC) have been reported in the literature, with an estimated incidence as low as 0.23%. This type of cancer is more prevalent among men aged 50–83 who are heavy smokers. NENs encompass paragangliomas and epithelial neoplasms. The latter categories include neuroendocrine tumors, or typical carcinoids, and NECs, or atypical carcinoids. Due to their nonspecific and often misleading presentation, and given the rarity of this condition, optimal management lacks standardization. Treatment typically involves a combination of surgery, chemotherapy, and radiotherapy. We present a case of supraglottic laryngeal NEC in a 61-year-old female nonsmoker. The patient underwent endoscopic excision followed by adjuvant radiotherapy.

## 1. Introduction

Neuroendocrine neoplasms (NENs) are uncommon in the head and neck region, with the larynx as the most frequent site [1]. To date, only 700 cases of laryngeal neuroendocrine carcinoma (NEC) have been reported in the literature [2]. The incidence of laryngeal NENs is estimated to be as low as 0.23% [3]. Due to its rarity, the presentation of laryngeal neuroendocrine tumors (NETs) is often nonspecific and can be misleading [3]. Optimal treatment for laryngeal NETs remains poorly documented. We present a case of a 61-year-old female, nonsmoker, diagnosed with NEC in the supraglottic region of the larynx. Treatment consisted of endoscopic excision followed by adjuvant radiotherapy (RT).

## 2. Case Report

A 61-year-old previously healthy, nonsmoking woman presented to our department with a 6-month history of dysphagia, dental infection, weight loss, and neck swelling. Dysphonia was absent. Physical examination revealed a palpable right Level II cervical lymph node (LN).

**Figure 1:**
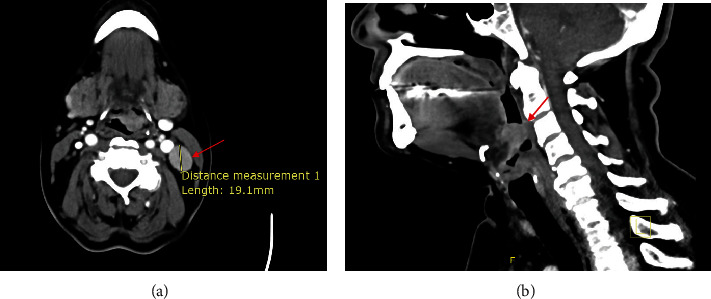
Computed tomography (CT) scan of the neck. (a) Axial view demonstrating left Level III cervical lymphadenopathy. (b) Sagittal view showing a lesion in the left supraglottic hemilarynx.

Fiber optic endoscopy demonstrated an exophytic mass on the left side of the epiglottis. Vocal cord mobility and pyriform sinuses appeared normal. The nasopharynx was clear. Neck computed tomography (CT) revealed a 22-mm, vascularized lesion in the left supraglottic larynx, invading the left epiglottic vallecula, aryepiglottic fold, and epiglottic free wall. Additionally, the CT scan showed bilateral Level III cervical lymphadenopathy, more prominent on the left side (maximum diameter: 19 mm), and multiple small supraclavicular LNs (Figure 1).

Our differential diagnosis included adenocarcinoma, acinic cell carcinoma, basaloid squamous cell carcinoma, solid type of adenoid cystic carcinoma, and poorly differentiated squamous cell carcinoma. A biopsy of the laryngeal mass suggested Grade 2, moderately differentiated NEC. An octreotide scan revealed no evidence of an octreotide-avid lesion elsewhere.

**Figure 2:**
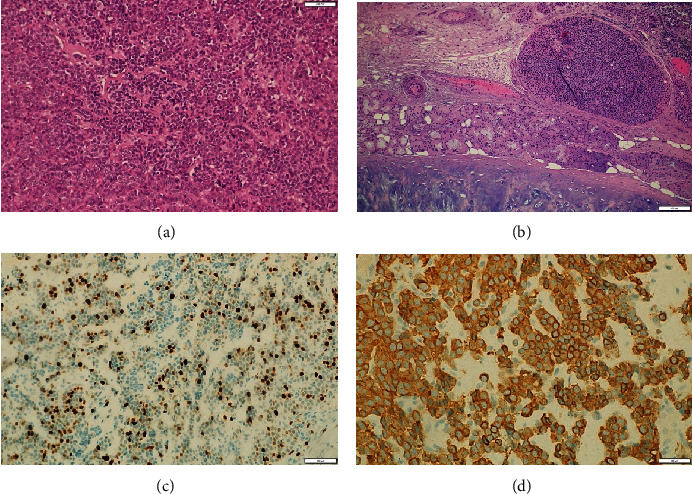
(a, b) Cancer cells. (c) Ki-67. (d) Chromogranin.

The patient underwent total epiglottic lesion excision and bilateral neck dissection. Histopathological findings confirmed moderately differentiated NEC (atypical carcinoid tumor), measuring 2.7 cm at its largest dimension. Immunohistochemistry showed a Ki-67 index of 40%. Chromogranin and synaptophysin staining were strongly positive and diffuse. Lymphovascular space invasion was present. Margins were clear of tumor. The mitotic count was up to 10 per high-power field (Figure 2). Twelve of the 104 harvested LNs exhibited metastasis with multiple extranodal extensions. The largest nodal metastatic deposit measured 3.5 cm. The patient was staged as pT2 N3b M0, Stage IVb.

Her case was discussed at a multidisciplinary tumor board, where adjuvant concurrent chemoradiotherapy was recommended. However, the patient declined chemotherapy. Therefore, she received adjuvant RT alone with doses of 66 and 60 Gy in 33 fractions, at 2 and 1.8 Gy per fraction, respectively. The patient completed treatment without significant side effects.

She has been followed for 3 years with normal voice quality, no dysphagia, and no evidence of disease recurrence on imaging or endoscopy.

## 3. Patient Simulation, Targets, and Organs-at-Risk (OARs) Delineation

CT simulation was performed with the patient in the supine position using an S-frame board and a thermoplastic mask for immobilization. CT simulation images were electronically transferred to the Eclipse (Varian Medical Systems, Palo Alto, CA) version 16.10.0 treatment planning system. These images were compared and fused with diagnostic CT scans.

Volumetric-modulated arc therapy plans were created for the TrueBeam linear accelerator (Varian Medical Systems, Palo Alto, CA) with jaw tracking enabled. The Photon Optimizer (ver. 16.1.0, Varian Medical Systems) was used for treatment plan optimization, and the anisotropic analytic algorithm (ver. 16.1.0, Varian Medical Systems) was used for dose calculation with a 1-mm grid.

The clinical target volume (CTV) and planning target volume (PTV) were delineated. The CTV was defined based on preoperative images and the initial tumor extent. A 5-mm margin was added in all directions to the CTV to create the PTV, accounting for patient setup and motion uncertainties.

OARs included the spinal cord, mandible, parotid glands, esophagus, oral cavity, thyroid, and lungs. A dose of 60 Gy in 33 fractions (1.8 Gy per fraction) was prescribed to the tumor bed and prophylactic LN stations, including bilateral Level I. Involved LN levels (bilateral Levels II and III) received 66 Gy in 33 fractions (2 Gy per fraction), with doses prescribed to the 90% isodose line.

**Figure 3:**
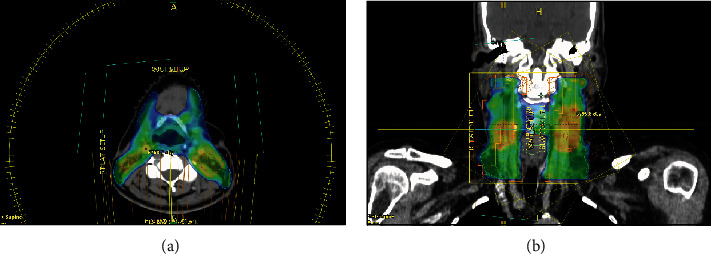
Dosimetric distribution. (a) Axial CT simulation cut showing a color wash for 95% of the PTV_60Gy. (b) Coronal view.

Daily cone-beam CT was performed for image verification. The PTV received a maximum dose of 66.7 Gy and a mean dose of 63.8 Gy (Figure 3). All dose constraints were met.

RT was well tolerated overall, with only Grade I dysphagia and dysgeusia reported by the patient.

## 4. Discussion

NENs are a heterogeneous group of malignancies originating from primitive stem cells. While predominantly found in the lungs and gastrointestinal tract, NENs can also arise in other organs, including the larynx, bronchus, liver, pancreas, kidneys, ovaries, prostate, and thymus [3]. Laryngeal NENs are rare, accounting for less than 1% of all laryngeal neoplasms, but they are the most common nonsquamous carcinoma of the larynx [4].

Since the first reported case by Rai in 1969 [5], approximately 700 cases of laryngeal NECs have been documented [2]. Early diagnosis is often delayed due to the rarity and nonspecific presentation of laryngeal NENs, leading to challenges in standardizing treatment. These tumors primarily affect the epiglottis and supraglottic regions, with other potential head and neck sites including the petrous apex, nasopharynx, tonsil, tongue, hypopharynx, and salivary glands [6].

While the condition is more prevalent among men aged 50–83 who smoke heavily [4], the present case involved an elderly, nonsmoking female. The clinical presentation of carcinoid tumors varies widely, from asymptomatic to the full spectrum of carcinoid syndrome, depending on tumor size and metastasis [3]. Common initial symptoms include hoarseness, dysphagia, and pain [7].

Diagnosis relies on identifying the characteristic neuroendocrine architecture and confirming neuroendocrine differentiation through immunohistochemistry, which typically shows positivity for chromogranin, synaptophysin, and cytokeratin [4]. Laryngeal NENs exhibit a broad pathological spectrum with diverse natural histories, treatments, and outcomes.

The World Health Organization (WHO) classification of NENs has evolved with advancements in pathological tools like Ki-67 and the mitotic count. A unified common International Agency for Research on Cancer/WHO terminology framework for all NENs, regardless of origin, has been proposed, categorizing epithelial NENs into NETs and NECs based on mitotic count (per square millimeters), proliferation indices (Ki-67 labeling index), and necrosis. NETs are further subdivided into G1, G2, and G3 based on proliferative features [8].

In the present study, a mitotic count of up to 10 per high-power field, Ki-67 of 40%, and diffuse positivity for chromogranin and synaptophysin led to a diagnosis of moderately differentiated or Grade II primary laryngeal NEC. Atypical carcinoid tumors are the most common type of laryngeal NEN, with a predilection for the supraglottic region [6]. Despite their slow growth, these tumors often exhibit aggressive behavior and high metastatic potential, affecting nearly 30% of patients at presentation [6]. An octreotide scan was performed in this case to evaluate for distal metastasis, which was negative.

Due to the rarity of laryngeal NENs, treatment guidelines are not well-established. Options include surgery, RT, and chemotherapy. For advanced NEC, chemotherapy, often combining cisplatin and etoposide with or without RT, is the primary treatment [1]. Unlike laryngeal small cell carcinoma, which is typically managed with concurrent chemoradiotherapy [7, 9], surgical excision is the mainstay treatment for localized laryngeal carcinoids, influenced by tumor size and extent. Given the high incidence of LN metastases, elective neck dissection is recommended [1]. Woodruff and Senie reported a 43% rate of nodal metastases in a series of 127 cases [10], while a meta-analysis of 436 patients demonstrated a 29.8% regional recurrence rate without neck dissection compared to 0% with neck dissection (*p* < 0.001) [6]. Elective neck dissection is therefore justified, typically involving bilateral Levels IIA and III for supraglottic tumors [2].

However, both local and distant recurrences are common, occurring in approximately 62.5% and 69.4% of cases, respectively, even after surgical resection [6]. This highlights the need for adjuvant therapies. Given the variable radiosensitivity of these tumors and their propensity for recurrence and metastasis, treatment strategies should consider the tumor subtype.

Postoperative RT is often used for atypical carcinoids, but its efficacy remains controversial due to limited evidence and conflicting results from small studies. A retrospective analysis of 127 atypical carcinoids found RT to be unsatisfactory [10], and a meta-analysis of 436 patients suggested no benefit from adjuvant RT. Patients treated with primary RT had lower disease-specific survival compared to those treated with surgery (53.8% vs. 60.2%; *p* = 0.035), while postoperative RT showed even worse outcomes (41.2%; *p* = 0.050) [6].

Nevertheless, a retrospective study by Gillenwater et al. at the University of Texas M.D. Anderson Cancer Center suggested potential benefits of RT in some patients with atypical carcinoid of the larynx [11], particularly those with local extension, multiple nodal metastases, or extracapsular disease [12]. In line with these recommendations, postoperative RT was indicated in the present case due to cervical LN metastases [13].

Despite the poor prognosis for atypical carcinoids, long-term follow-up is essential due to the risk of late recurrences. The study by Gillenwater et al. reported 5- and 10-year survival rates of 48% and 30%, respectively [11]. Palliative RT may be considered for symptomatic metastases to improve local control, alleviate symptoms, and maintain quality of life.

## 5. Conclusion

Laryngeal NENs are rare and heterogeneous malignancies with diverse pathological features, clinical courses, and prognoses. The rarity and complexity of these tumors have hindered the development of standardized treatment protocols. Current literature suggests that RT may improve locoregional control when administered after surgical resection for atypical carcinoids. However, definitive evidence supporting this practice is lacking and requires further investigation. For patients with localized advanced-stage disease unsuitable for surgery, RT, either alone or in combination with concurrent chemotherapy, is considered the preferred treatment approach.

In the palliative setting, RT is a valuable option for managing symptomatic metastases. This modality effectively slows disease progression, alleviates pain, and enhances the overall quality of life.

## Data Availability

The data supporting this case report are from previously reported studies and datasets, which have been cited. The processed data are available for the corresponding author upon request.
